# Self-reported questionnaire survey on the prevalence and symptoms of adverse food reactions in patients with chronic inhalant diseases in Tangshan city, China

**DOI:** 10.1186/s13223-017-0228-3

**Published:** 2018-02-02

**Authors:** Guodong Hao, Xuxin Lai, Zhijing Song, Zhixing Wang, Xing-ai Kong, Haifeng Zhong, Sui Fu Hui, Yiwu Zheng

**Affiliations:** 1 Department of Allergy, Tangshan Gongren Hospital, Tangshan, 063000 China; 2 Scientific Affairs, ALK, Guangzhou, 510300 China; 3 R606-607, No 5 Luoxuan 4th Road, International Bio-island, Guangzhou, 510300 China

**Keywords:** Adverse food reactions, Food allergy, Prevalence, Questionnaire, Symptom

## Abstract

**Background:**

The prevalence of adverse food reactions in patients with chronic inhalant diseases has seldom been studied in China. This study is to investigate the prevalence of adverse food reactions and the symptoms caused in respiratory patients.

**Methods:**

Respiratory patients in allergy clinics were asked to complete a questionnaire. Patients’ information such as age, gender, family history of allergy, and adverse reactions to a list of 48 foods and the symptoms caused, was recorded. Multivariate analyses were performed to determine the prevalence of adverse food reactions and their associated symptoms.

**Results:**

459 subjects, with an average age of 32 years old, completed the questionnaire; 45.3% were male. Among the 459 subjects, 38.1% (175/459) had an adverse reaction to food: 13.6% had an adverse food reaction to crab, 12.4% had an adverse food reaction to shrimp; and 9.9% had an adverse reaction to shellfish. Peach and nectarine were also shown to be common causative foods with 6.8% of the study group showing an adverse reaction to peach and 5.2% to nectarine. Seafood mainly caused skin symptoms and fruits gave rise to more throat, oral, and gastrointestinal problems.

**Conclusion:**

The prevalence of adverse food reactions is high for patients with respiratory diseases. This indicates that adverse food reactions should be considered in the treatment and management of patients with chronic inhalant diseases.

**Electronic supplementary material:**

The online version of this article (10.1186/s13223-017-0228-3) contains supplementary material, which is available to authorized users.

## Background

Food allergy (FA) is an IgE-mediated disease that negatively influences quality of life. In some cases, FA may cause life-threatening anaphylaxis. Up to 60% of patients with FA in older children, adolescents and adults have chronic inhalant diseases, such as asthma, rhinitis and conjunctivitis [1]. This was also seen in Chinese patients [2]. The prevalence of FA has increased dramatically during the past decades in developed countries. The prevalence of challenge-proved FA in infancy reached 10% [3]. Recent studies in developing countries showed a similar trend [4, 5]. It is anticipated that FA including FA-elicited anaphylaxis will soon become more prevalent [4]. Adverse food reactions (AFRs) are not clinically diagnosed as allergy but more commonly occur in both children and adults. The increasing prevalence of AFRs and FA induces serious public health problems, including medical costs, burden on families, and hospitalization.

In western countries, the majority of food allergies are caused primarily by eight foods, i.e. milk, egg, wheat, soy, peanut, tree nut, fish, and shellfish [6, 7]. These most common food allergens are prevalent in the diet of the modern lifestyle across the world. The epidemiologic studies of FA are relatively scarce in China. Most of the Chinese studies were conducted among the general population, especially among children [8–10] or infants [2, 11]. Patients with chronic inhalant diseases often have concomitant FA or AFRs. The prevalence of FA or AFRs among patients with chronic inhalant diseases has seldom been investigated in China.

In this study, we investigated the prevalence of AFRs and relevant symptoms in respiratory patients. We embarked on a questionnaire survey of self-reported AFRs in patients with chronic inhalant diseases. The study was performed in an allergy clinic in the city of Tangshan in Hebei Province, northern China.

## Methods

Subjects with a clinical history of chronic inhalant diseases such as rhinitis and/or asthma were enrolled in this study from January to December 2016. The patients were from the Department of Allergy, Tangshan Gongren Hospital. The subjects were asked to complete a self-administered questionnaire at face-to-face interviews. In addition to gathering information on age, gender and family history, the questionnaire was mainly used to collect information on AFRs to plant foods, sea foods, meat and other common foods, including cow’s milk and egg, in total 48 foods. The questionnaire also gathered information on the symptoms caused after the ingestion of certain foods referred to in the Japanese Guideline for Food Allergy 2014 [12]. The symptoms listed in the questionnaire were related to skin (urticaria, angioedema, eczema etc.), membrane (eye, nasal, and oral symptoms, including throat and tongue), respiratory organs (chest tightness, wheezing etc.), gastrointestinal discomfort or vomiting, drowsiness or dizziness and anaphylaxis. Information on treatment of AFRs and family history of allergy was also studied.

The study was approved by the Tangshan Gongren Hospital and all subjects provided a written informed consent form.

### Statistical analysis

The contingency analysis was used for the correlation between causative food and the relevant symptoms. The 48 kinds of food in the questionnaire were classified into 8 categories: fruit, nut, seafood, vegetable, grain, meat, egg, and milk. The 8 reported symptoms, including face/mouth; skin; throat; chest; gastrointestinal; nasal; eye; drowsiness/dizziness and anaphylaxis, were analyzed. The frequency of reported AFRs was summarized. The statistical analysis was performed in JMP^®^, Version 13.1 (SAS Institute Inc., Cary, NC).

## Results

This study recruited subjects with rhinitis and/or asthma who completed the questionnaire (Additional file 1) from the Allergy Department. From 2000 clinical patients with chronic inhalant disease, approximately 23% (459) were willing to participate in this study. The characteristics of participants are presented in Table 1. 45.3% are male, 21.4% are children (< 14 years old), and the mean age of participants was 32 years old. When recruited, the most common underlying condition was allergic rhinitis (81.2%). 32.5% of participants had asthma symptoms, and 31.2% also had skin symptoms (including urticarial, dermatitis, eczema). More than 34.2% of participants had a family history of allergy; among them 52.2% were their first-degree relatives (parents, siblings or children) and 37.6% did not specify which relatives were allergic.

**Table 1:** Characterization of subjects

Items	N (%)
Total subjects	2000
Self-reported subjects	459 (23)
Gender (male)	208 (45.3)
Children (< 14 years)	98 (21.4)
Age (mean)	32
Allergy family history	157 (34.2)
Symptoms at recruitment	
Asthma	149 (32.5)
Rhinitis	373 (81.2)
Asthma/rhinitis with hives, eczema	143 (31.2)

Among the 459 subjects, 38.1% (175/459) had an adverse reaction to foods. The majority of respondents (69.5%, 122/175) had AFRs against more than one single food. About 4.8% (22/459) of subjects displayed multiple reactions to more than 5 food items. 40.3% (185/459) of subjects reported AFRs to sea food, i.e. crab (13.6%), shrimp (12.4%) and shellfish (9.9%). The second most common foods to cause AFRs were peach and nectarine, which accounted for 13.5% (62/459) of subjects. The other notable causative foods were mango (4.9%), apple (4.3%), egg (3.3%), soybean (2.5%), and fish (2.5%), shown in Table 2.

**Table 2:** The causative food and corresponding prevalence of adverse food reactions

Food name	AFRs, N (%)
Crab	70 (13.6)
Shrimp	64 (12.4)
Shellfish	51 (9.9)
Peach	35 (6.8)
Nectarine	27 (5.2)
Mango	25 (4.8)
Apple	22 (4.3)
Egg	17 (3.3)
Soybean	13 (2.5)
Fish	13 (2.5)
Sunflower seed	12 (2.3)
Lychee	11 (2.1)
Tomato	11 (2.1)
Spicy flavoring	10 (1.9)
Cheery	9 (1.7)
Pineapple	9 (1.7)
Peanut	9 (1.7)
Kiwi	8 (1.6)
Apricot	7 (1.4)
Milk	6 (1.2)
Plum	5 (1.0)
Mutton	5 (1.0)
Chinese date	5 (1.0)
Strawberry	4 (0.8)
Banana	4 (0.8)
Beef	4 (0.8)
Dried longan	4 (0.8)
Alcohol	4 (0.8)
Grape	3 (0.6)
Melon	3 (0.6)
Chestnut	3 (0.6)
Walnut	3 (0.6)
Watermelon	3 (0.6)
Pear	2 (0.4)
Hazel	2 (0.4)
Cashew	2 (0.4)
Wheat	2 (0.4)
Carrot	2 (0.4)
Cucumber	2 (0.4)
Corn	2 (0.4)
Pork	2 (0.4)
Beer	2 (0.4)
Pine nut	1 (0.2)
Celery	1 (0.2)
Persimmon	1 (0.2)
Jackfruit	1 (0.2)
Other foods	15 (2.9)

Of the 175 respondents who reported having AFRs, 51.4% (90/175) had skin symptoms, such as eczema and urticaria; 40.0% (70/175) had face/mouth swelling or mouth itch; 33.1% (58/175) had throat tightness or discomfort. The percentage of subjects with chest, gastrointestinal, nasal and eye symptoms was 17.7, 13.7, 6.6, 2.3 and 1.1%, respectively, shown in Table 3. Among 175 subjects with AFRs, only 2 (1.1%) reported to have anaphylaxis. Moreover, both of them had adverse reactions to cherry and peach or nectarine. 5 subjects who had taken medicines due to AFRs and another 6 subjects had been hospitalized.

**Table 3:** The prevalence of self-reported symptoms due to adverse food reactions

Symptoms	Subjects N (%)
Skin issues, e.g. hives, eczema etc.	90 (51.4)
Face/mouth swelling or itch	70 (40.0)
Throat tightness or discomfort	58 (33.1)
Chest tightness or wheezing	31 (17.7)
Gastrointestinal discomfort or vomiting	24 (13.7)
Nasal sections or congestion	11 (6.6)
Eye itching or watering	4 (2.3)
Drowsiness or dizziness	2 (1.1)
Anaphylaxis	2 (1.1)

**Fig. 1:**
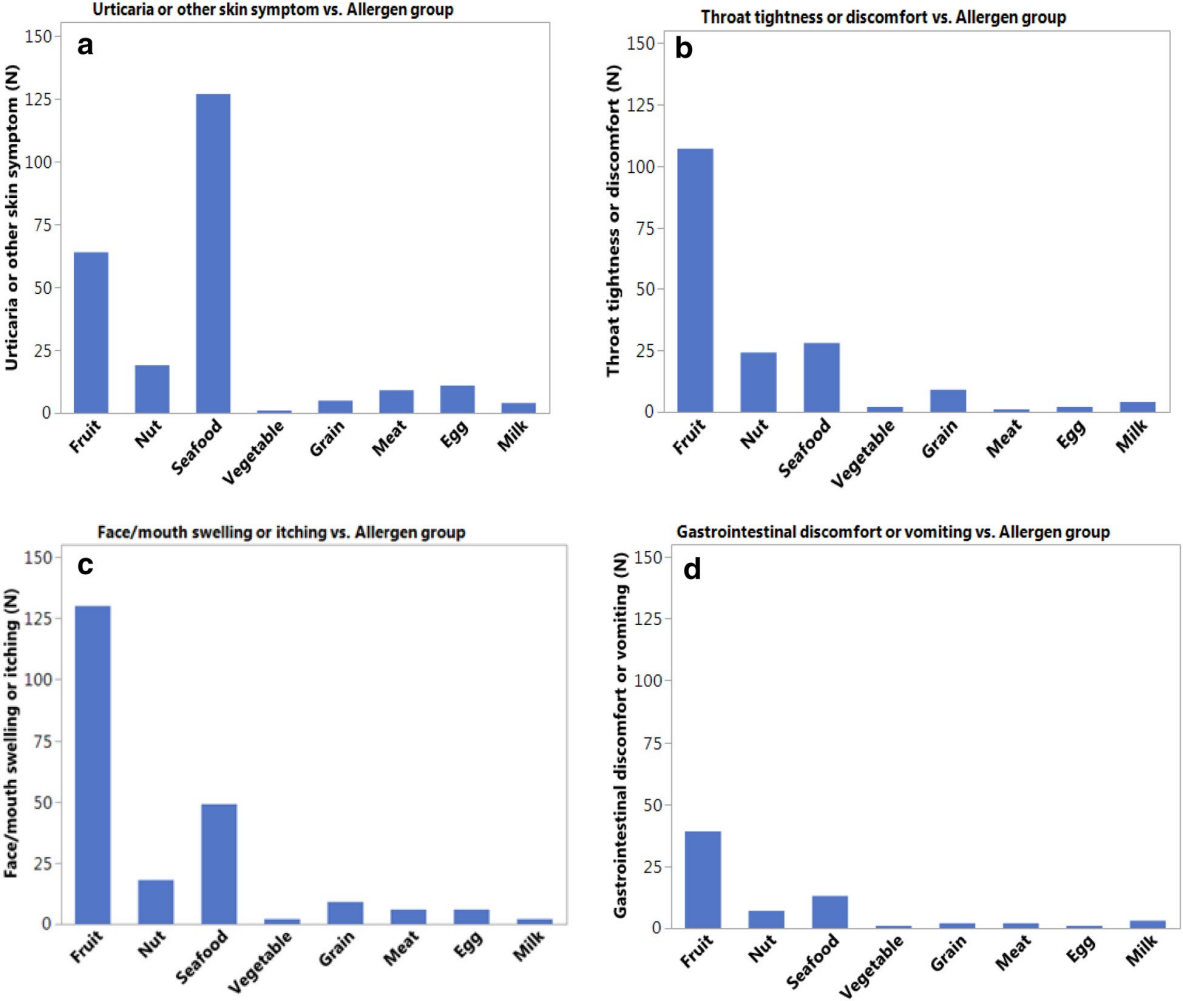
The correlations between food groups and relevant symptoms. **a** Skin symptoms are more likely associated to seafood; **b** Throat tightness or discomforts are more likely linked to fruits; **c** Face/mouth swelling or itch are more likely linked to fruits and seafood; **d** Gastrointestinal symptoms are mainly associated to fruits

The foods were divided into different groups, e.g. seafood, fruits, meats, nuts, vegetables, egg, milk etc. The associations between food groups and symptoms were analyzed. Skin symptoms were mainly associated with seafood, in contrast to throat tightness or discomfort, which was more likely to be linked to fruits. Gastrointestinal symptoms were mainly associated with fruits and seafood, but not milk or egg. Fruits and seafood were major triggers of face/mouth swelling and itch, see Fig. 1. AFRs triggered symptoms of chest tightness or wheezing more often than rhinitis symptoms. Other symptoms such as drowsiness and dizziness rarely occurred and the association with the responding food could not be analyzed. The symptoms caused by individual foods were similar to those caused by food groups (data not shown).

There were 41.4% of subjects with a family history of allergy reported AFRs, in contrast to 36.4% of subjects without a family history of allergy (p > 0.05).

## Discussion

In this study the prevalence of self-reported AFRs is high. It may be due to the fact that the subjects are patients with chronic inhalant diseases. Allergic diseases play an important role in the etiology of FA [13, 14]. The cross-reactivity between inhalant and food allergies was also an important contributor to the high prevalence, since subjects all had chronic inhalant diseases and sensitized to certain airborne allergens, e.g. house dust mites (HDM) and pollens. The prevalence and specific IgE level of HDM and weed pollens has been illustrated with subjects in another study at this clinic [15]. Our previous study at this hospital demonstrated a high correlation of specific IgE between birch pollen, soy and apple allergens [16]. Moreover, we used a self-reported questionnaire to estimate the prevalence of AFRs. It includes all AFRs both immune and non-immune in nature, e.g., lactose intolerance. Nwaru et al. [6] showed that self-reported FA was approximately six to ten times higher than the point prevalence of provocation-proven FA. A Turkish study of 3500 school children showed that parent-reported FA was more than 7 times higher than the prevalence confirmed by open food challenge (OFC) [17]. A study in China with OFC revealed an FA prevalence rate of 4.0% in Chongqing city in infants of 0–12 months old, [11] which was almost ten times lower than the AFRs prevalence in this study. On the other hand, most subjects (78.6%) in this study were adults (> 14 years) and the prevalence of FA or AFRs should be lower than in young children, as the incidence of AFRs is normally higher in children than in adults.

We demonstrated that shellfish is the most common causative food in this work. Other studies have also demonstrated that shellfish is the most prevalent FA among the Chinese population [8–10] even in infants [11, 18]. The most common FA in children under 5 years old is relatively similar across the world including milk, egg, peanuts and seafood. Chinese studies [5, 18, 19] also illustrated that egg is the most important food allergen in infants and caused skin and gastrointestinal symptoms [5]. Egg and milk allergies were not prevalent in this work, mainly because milk and egg allergies occur in younger age groups rather than in older, while the prevalence of crustacean, fish and plant food allergy were higher in older than in younger [7]. Furthermore, food tolerance to allergens in egg and milk is more likely to be acquired with age, than developing tolerance to allergens in crustacean, fish and plant foods [12]. Other common causative foods such as peanuts, nuts, and seeds are prevalent FAs in Europe and the United States [7], but were not prevalent in this study.

Fruit allergens such as apple and kiwi have been consistently shown to be common allergens in many European countries [20]. This study highlighted that peach, nectarine, and mango were the most common causative fruits of AFRs in China. This may be due to a difference in geographic location. Other AFRs to meat (beef and pork), grain (including wheat), and vegetables did not occur frequently. Some unusual causative foods, such as silkworm chrysalis and cicada were reported in this work, although it may be due to a cross-reaction to shellfish proteins such as tropomyosin [21].

Symptoms of FA may involve single or multiple organs with the severity ranging from mild and local to life-threatening anaphylaxis [1]. Oral allergy syndrome (OAS) [22] is the most frequent clinical presentation of FA in adolescent and adult patients and causes itch, swelling or discomfort of the lips, tongue, palate, throat, and ears following food ingestion. OAS can be elicited by any food and often occurs in pollen-allergic patients with cross-reactivity to fruits and nuts. FA patients to fruits without concomitant pollen allergy usually trigger more severe symptoms [23]. In this study, oral symptoms were the second-most common signs. Fruits such as peach, nectarine, and mango were the most causative foods to trigger oral problems. After the oral mucosa, skin is an important target organ involved in FA and elicits symptoms such as urticarial, atopic dermatitis, eczema, etc. [1]. The most common symptom induced in this study was skin problems mainly led by adverse reactions to seafood. Gastrointestinal allergy mainly occurs in infancy and the most common causative food is cow’s milk. It illustrated that up to 70% of patients develop a tolerance to milk at 1 year of age and approximately 90% develop tolerance at 2 years old [12]. The youngest subjects in this study were 4 years old. Gastrointestinal symptoms were not prevalent and mainly elicited by fruit and seafood. Respiratory symptoms were rarely observed as the sole manifestation of FA, but were more frequently linked to reactions to other organs. Anaphylactic reactions were reported by two patients in this study. One suffered from asthma and AFRs and one had diagnostic FA-elicited angioedema and urticaria. Severe anaphylaxis is more likely to occur in patients with FA accompanied by asthma [12].

In conclusion, the prevalence of self-reported AFRs in patients with chronic inhalant diseases is high for Chinese adolescents and adults. The most common causative foods are seafood and peaches. Seafood mainly causes skin symptoms and fruit gives rise to more oral problems. Respiratory symptoms are also triggered in adverse food reactions. It indicates that adverse food reactions should be considered in the treatment and management of patients with chronic inhalant diseases.

## Additional file

**Additional file 1.** Self-reported questionnaire survey on the prevalence and symptoms of food adverse reactions in patients with asthma, rhinitis and conjunctivitis. The questionnaire was developed referring to [1].

## References

[1] Werfel T, Asero R, Baller-Weber BK, Beyer K, Enrique E, Knulst AC (2015). Position paper of the EAACI: food allergy due to immunological cross-reactions with common inhalant allergens. Allergy.

[2] Gao Q, Ren YX, Liu YG, Ma L, Gu XH, Zhang WX (2017). Allergy march of Chinese children with infantile allergic symptoms: a prospective multi-center study. World J Pediatr..

[3] Osborne NJ, Koplin JJ, Martin PE, Gurrin LC, Lowe AJ, Matheson MC, HealthNuts Investigators (2011). Prevalence of challenge-proven IgE-mediated food allergy using population-based sampling and predetermined challenge criteria in infants. J Allergy Clin Immunol.

[4] Prescott SL, Pawankar R, Allen KJ, Campbell DE, Sinn JKh, Fiocchi A (2013). A global survey of changing patterns of food allergy burden in children. World Allergy Organ J.

[5] Hu Y, Chen J, Li H (2010). Comparison of food allergy prevalence among Chinese infants in Chongqing, 2009 versus 1999. Pediatr Int.

[6] Allen JK, Kopli JJ (2012). The epidemiology of IgE-mediated food allergy and anaphylaxis. Immunol Allergy Clin North Am.

[7] Nwaru BI, Hickstein L, Panesar SS, Roberts G, Muraro A, Sheikh A, EAACI food Allergy & Anaphylaxis Guidelines Group (2014). Prevalence of common food allergies in Europe: a systematic review and meta-analysis. Allergy.

[8] Yang Z, Zheng W, Yung E, Zhong N, Wong GW, Li J (2015). Frequency of food group consumption and risk of allergic disease and sensitization in schoolchildren in urban and rural China. Clin Exp Allergy.

[9] Zeng GQ, Luo JY, Huang HM, Zheng PY, Luo WT, Wei NL (2015). Food allergy and related factors in 2540 preschool children: an epidemiological survey in Guangdong Province, southern China. World J Pediatr..

[10] Zhang Y, Chen Y, Zhao A, Li H, Mu Z, Zhang Y (2015). Prevalence of self-reported food allergy and food intolerance and their associated factor in 3–12 year-old children in 9 areas in China. Wei Sheng Yan Jiu.

[11] Chen J, Hu Y, Allen KJ, Ho MH, Li H (2011). The prevalence of food allergy in infants in Chongqing, China. Pediatr Allergy Immunol.

[12] Urisu A, Ebisawa M, Ito K, Aihara Y, Ito S, Mayumi M, Committee for Japanese Pediatric Guideline for Food Allergy, Japanese Society of Pediatric Allergy and Clinical Immunology and Japanese Society of Allergology (2014). Japanese guideline for food allergy 2014. Allergol Int..

[13] Virkud YV, Burks AW, Steele PH, Edwards LJ, Berglund JP, Jones SM (2017). Novel baseline predictors of adverse events during oral immunotherapy in children with peanut allergy. J Allergy Clin Immunol.

[14] Muraro A, Werfel T, Hoffmann-Sommergruber K, Roberts G, Beyer K, Bindslev-Jensen C, EAACI Food Allergy and Anaphylaxis Guideline Group (2014). EAACI food allergy and anaphylaxis guidelines: diagnosis and management of food allergy. Allergy.

[15] Hao G, Zheng Y, Gjesing B, Kong X, Wang J, Song Z (2013). Prevalence of sensitization to weed pollens of *Humulus scanderns*, *Artemisia vulgaris*, and *Ambrosia artemisiifolia* in northern China. J Zhejiang Uni-Sci B..

[16] Hao G, Zheng Y, Wang Z, Kong X, Song Z, Lai X (2016). High correlation of specific IgE sensitization between birch pollen, soy and apple allergens indicates pollen-food allergy syndrome among birch pollen allergic patients in northern China. J Zhejiang Univ-Sci B..

[17] Orhan F, Karakas T, Cakir M, Aksoy A, Baki A, Gedik Y (2009). Prevalence of immunoglobulin E-mediated food allergy in 6–9-year-old urban schoolchildren in the eastern Black Sea region of Turkey. Clin Exp Allergy.

[18] Liu FL, Ning YB, Ma DF, Zheng YD, Yang XG, Li WJ (2013). Prevalence of self-reported allergy, food hypersensitivity and food intolerance and their influencing factors in 0–36 months old infants in 8 cities in china. Zhonghua Er Ke Za Zhi.

[19] Chen J, Liao Y, Zhang HZ, Zhao H, Chen J, Li HQ (2012). Prevalence of food allergy in children under 2 years of age in three cities in China. Zhonghua Er Ke Za Zhi.

[20] Steinke M, Fiocchi A, Kirchlechner V, Baller-Weber B, Brockow K, Hischenhuber C, REDALL study consortium (2007). Perceived food allergy in children in 10 European nations. A randomized telephone survey. Int Arch Allergy Immunol.

[21] Faber MA, Pascal M, EI Kharbouchi O, Sabato V, Hagendorens MM, Decuyper (2017). Shellfish allergens: tropomyosin and beyond. Allergy.

[22] Mari A, Ballmer-Weber BK, Vieths S (2005). The oral allergy syndrome: improved diagnostic and treatment methods. Curr Opin Allery Clin Immunol.

[23] Fernandez-Rivas M, Bolhaar S, Gonzalez-Mancebo E, Asero R, van Leeuwen A, Bohle B (2006). Apple allergen across Europe: how allergen sensitization profiles determine the clinical expression of allergies to plant foods. J Allergy Clin Immunol.

