# Supplementary material for: Self-reported questionnaire survey on the prevalence and symptoms of adverse food reactions in patients with chronic inhalant diseases in Tangshan city, China
Source: Allergy Asthma Clin Immunol. 2018 Feb 2;14:3. doi: 10.1186/s13223-017-0228-3 (PMC5796569; doi:10.1186/s13223-017-0228-3)
Supplement: Supplementary file 1 — Additional file 1. Self-reported questionnaire survey on the prevalence and symptoms of food adverse reactions in patients with asthma, rhinitis and conjunctivitis. The questionnaire was developed referring to [1]. [file 13223_2017_228_MOESM1_ESM.docx]

**Self-reported questionnaire survey on the prevalence and symptoms of food adverse reactions in patients with asthma, rhinitis and conjunctivitis**

Date： _____________________

Name: Guardian:

Mobile: ­­­­­­­­ Address:

Birth: Gender:

SPT: Serum sample:

Diagnosis: asthma, rhinitis, conjunctivitis, others: ______________________

Disease history: Family allergy history:

| Food | Influence on QoL* | | | Symptom | | | | | | | | | Treatment | | |
| --- | --- | --- | --- | --- | --- | --- | --- | --- | --- | --- | --- | --- | --- | --- | --- |
|  | little | normal | serious | Face/ mouth/ itch welling | Throat/ tongue  tightness, itch, swelling | Chest tightness, wheezing | Atopic dermatitis urticaria, eczema, et. | Drowsiness,  Dizziness | Gastrointestinal discomfort,  vomiting,  pain | Nasal sections, congestion, itch | Eye red, itch, swelling | A* | M* | H* | N* |
| Apple |  |  |  |  |  |  |  |  |  |  |  |  |  |  |  |
| Cherry |  |  |  |  |  |  |  |  |  |  |  |  |  |  |  |
| Nectarine |  |  |  |  |  |  |  |  |  |  |  |  |  |  |  |
| Peach |  |  |  |  |  |  |  |  |  |  |  |  |  |  |  |
| Pear |  |  |  |  |  |  |  |  |  |  |  |  |  |  |  |
| Apricot |  |  |  |  |  |  |  |  |  |  |  |  |  |  |  |
| Plum |  |  |  |  |  |  |  |  |  |  |  |  |  |  |  |
| Kiwi |  |  |  |  |  |  |  |  |  |  |  |  |  |  |  |
| Strawberry |  |  |  |  |  |  |  |  |  |  |  |  |  |  |  |
| Persimmon |  |  |  |  |  |  |  |  |  |  |  |  |  |  |  |
| Jackfruit |  |  |  |  |  |  |  |  |  |  |  |  |  |  |  |
| Lychee |  |  |  |  |  |  |  |  |  |  |  |  |  |  |  |
| Mango |  |  |  |  |  |  |  |  |  |  |  |  |  |  |  |
| Grape |  |  |  |  |  |  |  |  |  |  |  |  |  |  |  |
| Pineapple |  |  |  |  |  |  |  |  |  |  |  |  |  |  |  |
| Banana |  |  |  |  |  |  |  |  |  |  |  |  |  |  |  |
| Melon |  |  |  |  |  |  |  |  |  |  |  |  |  |  |  |
| Tomato |  |  |  |  |  |  |  |  |  |  |  |  |  |  |  |
| Fig |  |  |  |  |  |  |  |  |  |  |  |  |  |  |  |
| Chestnut |  |  |  |  |  |  |  |  |  |  |  |  |  |  |  |
| Sunflower seed |  |  |  |  |  |  |  |  |  |  |  |  |  |  |  |
| Hazel |  |  |  |  |  |  |  |  |  |  |  |  |  |  |  |
| Peanut |  |  |  |  |  |  |  |  |  |  |  |  |  |  |  |
| Pine nut |  |  |  |  |  |  |  |  |  |  |  |  |  |  |  |
| Cashew |  |  |  |  |  |  |  |  |  |  |  |  |  |  |  |
| Walnut |  |  |  |  |  |  |  |  |  |  |  |  |  |  |  |
| Wheat |  |  |  |  |  |  |  |  |  |  |  |  |  |  |  |
| Soybean |  |  |  |  |  |  |  |  |  |  |  |  |  |  |  |
| Carrot |  |  |  |  |  |  |  |  |  |  |  |  |  |  |  |
| Celery |  |  |  |  |  |  |  |  |  |  |  |  |  |  |  |
| Cucumber |  |  |  |  |  |  |  |  |  |  |  |  |  |  |  |
| Potato |  |  |  |  |  |  |  |  |  |  |  |  |  |  |  |
| Corn |  |  |  |  |  |  |  |  |  |  |  |  |  |  |  |
| Egg |  |  |  |  |  |  |  |  |  |  |  |  |  |  |  |
| Shellfish |  |  |  |  |  |  |  |  |  |  |  |  |  |  |  |
| Shrimp |  |  |  |  |  |  |  |  |  |  |  |  |  |  |  |
| Crab |  |  |  |  |  |  |  |  |  |  |  |  |  |  |  |
| Milk |  |  |  |  |  |  |  |  |  |  |  |  |  |  |  |
| Pork |  |  |  |  |  |  |  |  |  |  |  |  |  |  |  |
| Lamb |  |  |  |  |  |  |  |  |  |  |  |  |  |  |  |
| Beef |  |  |  |  |  |  |  |  |  |  |  |  |  |  |  |
| Beer |  |  |  |  |  |  |  |  |  |  |  |  |  |  |  |
| Sea fish |  |  |  |  |  |  |  |  |  |  |  |  |  |  |  |
| watermelon |  |  |  |  |  |  |  |  |  |  |  |  |  |  |  |
| Date |  |  |  |  |  |  |  |  |  |  |  |  |  |  |  |
| Dried longan |  |  |  |  |  |  |  |  |  |  |  |  |  |  |  |
| Alcohol |  |  |  |  |  |  |  |  |  |  |  |  |  |  |  |
| Spicy flavoring |  |  |  |  |  |  |  |  |  |  |  |  |  |  |  |

^QoL: quality of life; A: anaphylaxis; M: medicine; H: hospitalization; N: None^
